# The Role of Placental Growth Factor in the Prediction of Carbohydrate and Thyroid Disorders during Pregnancy

**DOI:** 10.3390/medicina58020232

**Published:** 2022-02-03

**Authors:** Vesselina Yanachkova, Radiana Staynova, Emilia Naseva, Zdravko Kamenov

**Affiliations:** 1 Department of Endocrinology, Specialized Hospital for Active Treatment of Obstetrics and Gynaecology “Dr Shterev”, 1330 Sofia, Bulgaria; 2 Department of Pharmaceutical Sciences, Faculty of Pharmacy, Medical University of Plovdiv, 4002 Plovdiv, Bulgaria; radiana.staynova@mu-plovdiv.bg; 3 Department of Health Economics, Faculty of Public Health “Prof. Tsekomir Vodenicharov, MD, DSc”, Medical University of Sofia, 1527 Sofia, Bulgaria; emilia.naseva@gmail.com; 4 Department of Internal Medicine, Medical University of Sofia, 1431 Sofia, Bulgaria; zkamenov@hotmail.com; 5 Clinic of Endocrinology, University Hospital “Alexandrovska”, 1431 Sofia, Bulgaria

**Keywords:** pregnancy, placental growth factor, endocrine disorders

## Abstract

*Background and objectives:* To assess whether placental growth factor (PlGF) levels may have a predictive value for the onset of gestational diabetes mellitus (GDM) and thyroid dysfunction during pregnancy. *Materials and Methods:* This single-center retrospective analysis was conducted at the Specialized Hospital for Active Treatment in Obstetrics and Gynecology “Dr. Shterev”, Sofia, Bulgaria, from December 2017 to December 2019. Using pregnant women’s electronic records, we analyzed and compared the data of 412 women diagnosed with GDM and 250 women without evidence for carbohydrate disorders. Thyroid function was tested in all patients at the time of performing GDM screening. The following measurements were compared and assessed: body mass index (BMI), fasting blood glucose levels, thyroid-stimulating hormone levels (TSH), free thyroxine, and triiodothyronine (FT4 and FT3) levels, and serum placental growth factor (PlGF). The sensitivity and specificity of PlGF as a predictive marker for GDM and thyroid dysfunction were analyzed using receiver operating characteristic (ROC) curves. *Results:* There were no significant differences between GDM and control groups in terms of age and BMI (*p* > 0.05). In patients with established GDM, the PlGF corrected multiple of the median (MoM) was significantly higher compared to the control group (0.9 vs. 0.7, *p* < 0.001). The ROC-AUC for the prediction of GDM and thyroid dysfunction during pregnancy was 0.68 (95% CI 0.64–0.72) and 0.61 (95% CI 0.57–0.65), respectively. *Conclusions:* Our results underscore the potential role of PlGF as a biomarker in the prediction and diagnosis of GDM and thyroid dysfunction during pregnancy.

## 1. Introduction

Thyroid dysfunction and gestational diabetes mellitus (GDM) are the two most common endocrine disorders that can be registered during pregnancy. These conditions are at the root of several complications for both the mother and the fetus [1,2,3]. Therefore, adequate follow-up of pregnant women and timely diagnosis of thyroid and carbohydrate impairments is essential.

Although pregnancy is a physiological condition, it may change the way thyroid hormone production and glucose homeostasis are balanced. To some extent, this is associated with the development of the placenta and the secretion of a number of hormones, growth factors, and enzymes [4].

In most pregnant women, the adaptation mechanisms of thyroid gland function and carbohydrate metabolism provide the necessary hormonal and metabolic terms for the normal course of pregnancy. In some pregnant women, however, the production of thyroid hormones does not lead to the necessary adaptation to the requirements of pregnancy. This could be related to the development of subclinical or overt hypothyroidism during pregnancy. Given the role of the normal levels of thyroid hormones in carbohydrate metabolism, this could be associated with possible disorders in the latter [5].

Universal screening for thyroid dysfunction in early pregnancy has not been widely recommended by scientific societies. The ongoing debate towards universal screening is based on whether the treatment of women identified with thyroid disease, in particular subclinical hypothyroidism, is beneficial and cost-effective. Most of the current guidelines recommend targeted or selective screening. However, in this case, mainly women with risk factors for thyroid dysfunction are tested. Thyroid status is tested in pregnant women with concomitant thyroid disease or those with known risk factors such as a family history of thyroid disease, those living in an endemic area with iodine deficiency, goiters, positive antithyroid antibodies, dyslipidemia, type 1 diabetes, other autoimmune diseases, obesity, infertility, premature birth, etc. [6,7,8,9]. This selective screening fails to detect thyroid dysfunction in women without risk factors. It is well known that thyroid hormones are essential for the normal course of pregnancy and the normal development of the fetus, in particular its nervous system. Therefore, early detection and elimination of abnormalities in thyroid hormone levels are of great importance.

Regarding carbohydrate metabolism, universal screening for GDM is recommended in the period between 24–28 weeks of gestation [10,11,12]. In many cases, this is a prerequisite for delaying the diagnosis, and it is often facilitated after complications have already occurred.

The question arises whether biochemical markers of placentation, such as placental growth factor (PlGF), may have some predictive value in the early diagnosis of GDM and/or thyroid dysfunction.

During pregnancy, the main focus is on the placenta, which can be considered as a large temporary endocrine organ. The placenta produces a variety of hormones such as human placental lactogen, human chorionic gonadotropin (hCG), progesterone, estradiol, somatotropic hormone, cortisol, placental growth factor (PlGF), soluble FMS-like tyrosine kinase 1 (sflt-1), pregnancy-associated plasma protein-A (PAPP-A), prolactin, cytokines, and others [5]. Most of these hormones contribute to the occurrence of pregnancy-associated insulin resistance (IR) [13,14]. Pre-existing overweight and obesity, as well as excessive weight gain during pregnancy, are associated with the deepening of the IR [15]. It is well known that IR could become a prerequisite for the manifestation of carbohydrate impairments during pregnancy [14]. Placental hormones may also affect thyroid function. The effects of hCG on the thyroid gland have long been known [16]. In recent years, the effect that PlGF can have on maternal thyroid function was discussed [17,18].

Taken separately, most placental products show attitudes towards changes in insulin sensitivity and carbohydrate metabolism, respectively, mainly due to their insulin-antagonistic effects. Each of them can have its prognostic value for the manifestation of GDM. The only factor that is associated not only with carbohydrate metabolism but also with thyroid function is PlGF. Therefore, the present analysis considers the possibility of assessing its predictive role in the manifestation of the two most common endocrine disorders during pregnancy.

## 2. Materials and Methods

### 2.1. Study Setting and Patients

This single-center retrospective analysis was conducted at the Specialized Hospital for Active Treatment in Obstetrics and Gynecology “Dr. Shterev”, Sofia, Bulgaria, from January 2017 to January 2019. Using pregnant women’s electronic records, we analyzed and compared the data of 412 women diagnosed with GDM (case group) and 250 women without evidence of carbohydrate disorders (control group). Only the data of pregnant women with singleton pregnancies were used for the analysis. Women in the age range of 18 to 40 years without evidence of impairments in carbohydrate metabolism or thyroid dysfunction before pregnancy were included in the observation. This study population was drawn from our previously conducted study assessing the relationship between GDM and thyroid dysfunction [19].

### 2.2. Measurements

The diagnostic criteria for GDM were based on the International Association of the Diabetes and Pregnancy Study Groups recommendations, using the one-step strategy and performing a 2-h 75-g oral glucose tolerance test (OGTT) [20]. Thyroid function was tested in all patients at the time of performing GDM screening. The criteria of the European thyroid association guidelines for the management of subclinical hypothyroidism in pregnancy were used to determine thyroid dysfunction [9].

The following measurements were compared and assessed: body mass index (BMI), fasting blood glucose levels, thyroid-stimulating hormone levels (TSH), free thyroxine, and triiodothyronine (FT4 and FT3) levels and serum PlGF.

BMI was calculated by dividing a pregnant woman’s weight (in kilograms) by the square of her height (in meters). Fasting blood glucose was determined by the hexokinase method.

TSH, FT4, and FT3 levels were measured by an immunochemiluminescent method (Cobas 6000, Roche Diagnostics). Serum PlGF was tested in the period between 11–13 weeks of gestation in favor of aneuploid screening. PlGF levels were measured using an electro-chemiluminescent method ECLIA (Cobas 8000, Roche Diagnostics) and presented as multiples of the median (MoM) after correction based on maternal characteristics and medical history (Software provided by the Fetal Medicine Foundation London).

### 2.3. Data Analysis

SPSS software, version 24.0 (SPSS Inc., Chicago, IL, USA), was used for all data analyses. The Kolmogorov–Smirnov and Shapiro–Wilk tests were used to assess whether the continuous variables were normally distributed. The Mann–Whitney U-test was used to compare differences between the GDM and control groups. Continuous variables are presented as a mean (±standard deviation) or median and interquartile range (IQR). The sensitivity and specificity of PlGF as a predictive marker for GDM and thyroid dysfunction were analyzed using receiver operating characteristic (ROC) curves, and the area under the curves (AUC) was calculated. The cut-point is chosen at the maximum Youden index (J = sensitivity + specificity-1). A *p*-value of < 0.05 was considered statistically significant.

## 3. Results

Maternal characteristics and biochemical parameters of observed women are shown in Table 1.

There were no significant differences between GDM and control groups in terms of age and BMI (*p* > 0.05). The fasting blood glucose levels were significantly higher in patients with established GDM (*p* < 0.001). Significant differences regarding TSH, FT4, and FT3 levels were observed between the two groups of women (Table 1).

In patients with established GDM, the PlGF corrected multiple of the median (MoM) was significantly higher compared to the control group (0.9 vs. 0.7, *p* < 0.001).

**Figure 1:**
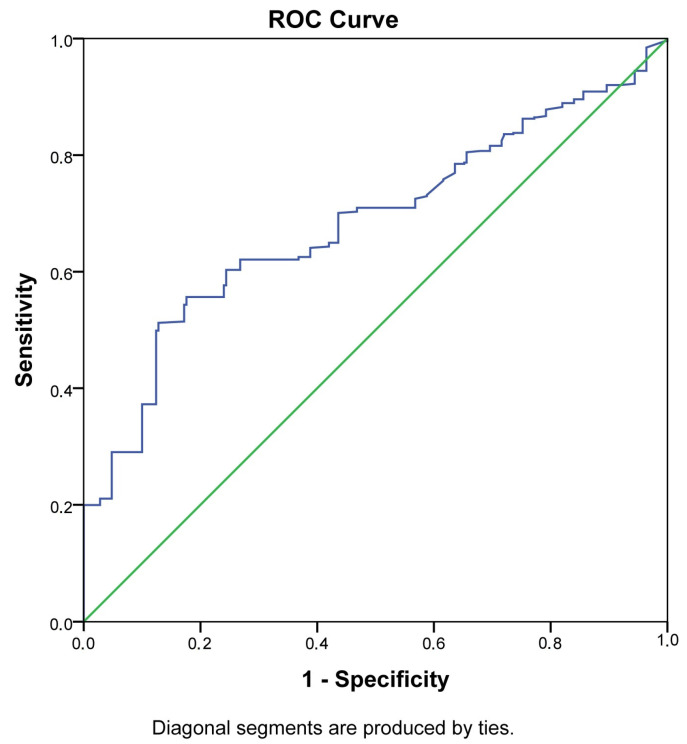
ROC curve of PlGF in the prediction and diagnosis of GDM.

**Figure 2:**
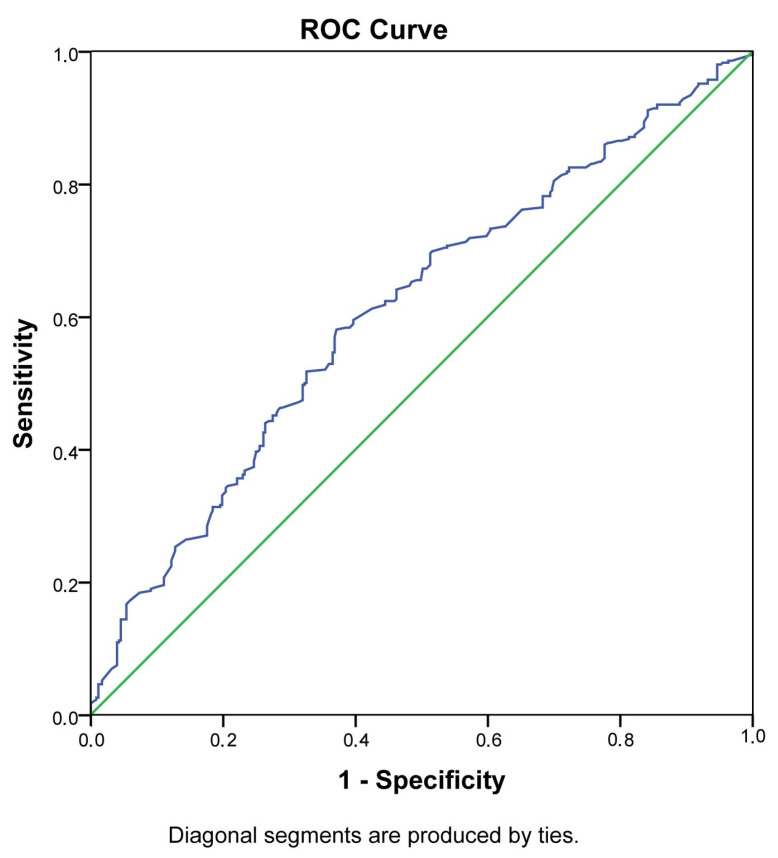
ROC curve of PlGF in the prediction and diagnosis of thyroid dysfunction.

The potential role of PlGF as a diagnostic marker for thyroid dysfunction and GDM was evaluated by ROC analysis (Figure 1 and Figure 2).

The AUC for PlGF as a predictive marker for GDM was 0.68 (95% CI 0.64–0.72, *p* < 0.001) and shows a moderately high discriminant capacity (Table 2). The best predictive value for GDM was observed at PlGF MoM from 0.89 upwards (with a relatively low sensitivity of 51.2% and higher specificity of 87.2%).

The AUC for PlGF as a predictive marker for thyroid dysfunction was 0.61 (95% CI 0.57–0.65, *p* < 0.001) and shows a medium–high discriminative ability (Table 3). The best predictive value for thyroid dysfunction was observed at a PlGF MoM of 0.81 upwards (with a relatively low sensitivity of 58.2% and slightly higher specificity of 62.9%).

## 4. Discussion

In modern screening for aneuploidies, which is performed at the end of the first trimester of pregnancy, in addition to hCG, s-flT-1, and PAPP-A, PlGF is also used as a marker. If some of these placental factors (hCG, PlGF, s-flT-1) can be associated with changes in thyroid function, there is evidence that PlGF may also be involved in carbohydrate disorders manifested during pregnancy [17,18,21,22].

PlGF belongs to the vascular endothelial growth factor (VEGF) family. It is expressed mainly in the placenta. Low levels of PlGF were also registered in other tissues—thyroid gland, heart, lungs, liver, skeletal muscle, and bone [23]. In the syncytiotrophoblast and cytotrophoblast of the placenta, PlGF induces the proliferation, migration, and activation of endothelial cells. This determines its major role in the development of placental vascularization [24]. Circulating PlGF levels are markedly elevated during pregnancy. In the human placenta, during the different stages of its development, the expression of PlGF is different. During the first trimester of an uncomplicated pregnancy, PlGF concentrations are low, with an increase from 11 to 12 weeks, and a peek at 30 weeks of gestation, followed by floating in its levels. Therefore, the placental expression of PlGF predominates after the second trimester when uteroplacental circulation progresses. This is manifested by altered angiogenesis of fetoplacental circulation and altered maturation of uteroplacental circulation, coinciding with increased expression of PlGF in later pregnancy. PlGF increases the proliferation of trophoblast cells and also reduces their apoptosis. Therefore, the development of placental circulation is influenced by PlGF [23].

Studies have shown that the PlGF is one of the main predictors of the occurrence of pregnancy complications such as pre-eclampsia and intrauterine growth retardation [25,26,27].

Although PlGF has been identified as a proangiogenic factor, it has been found that an excess of it can lead to an antiangiogenic effect. Therefore, it can be assumed that PlGF may have a direct effect on the thyroid gland [17,18]. Changes in glandular vascularization and its perfusion may lead to an impaired thyroid response to hCG. A study conducted by Korevaar et al. showed that elevated PIGF levels were associated with lower FT4 levels, and hence the approximately two-fold higher probability of hypothyroxinemia [17]. The effects were more pronounced in antibody (anti-TPO) positive women. Early high levels of this factor may be associated with the risk of maternal thyroid dysfunction [17,18].

As we already mentioned, PlGF belongs to the family of vascular endothelial growth factors. In patients with type 1 and type 2 diabetes mellitus, its increased expression is associated with retinal neovascularization and the manifestation of proliferative diabetic retinopathy. In published studies, an increase in PlGF was observed in pregnant women with GDM [21,22,28,29]. Eleftheriades et al. found that there were increased levels of PLGF in pregnancies complicated by GDM [28]. The explanation comes from the fact that hyperglycemia affects angiogenesis and maternal hyperglycemia and stimulates placental neovascularization. Changes in PlGF levels are primarily positively associated with fasting blood glucose levels. This may explain its predictive role in women who do not have risk factors for diabetes or pre-pregnancy insulin resistance [29,30,31].

Therefore, PlGF may alter thyroid function and, on the other hand, affect carbohydrate metabolism.

The disorders of thyroid function are associated with changes in carbohydrate metabolism and the manifestation of IR. Thus, thyroid disease and GDM, in turn, can be linked in a pathophysiological aspect. These relationships have their significance and corresponding consequences related to insulin needs and insulin sensitivity. Unrecognized thyroid dysfunction may impair glycemic control. A link has been established between thyroid hormones and the basal mechanisms that control appetite and energy expenditure, and this is ultimately associated with changes in insulin sensitivity [32].

Pre-existing maternal obesity, as well as excessive weight gain during pregnancy, are associated with the deepening of the IR, which becomes a prerequisite for the manifestation of carbohydrate abnormalities. On the other hand, obesity leads to increased peripheral deiodinase activity. This changes the ratio of free thyroid hormones FT3: FT4 (triiodothyronine: tetraiodothyronine) in favor of FT3, which is the biologically active hormone. Changes in thyroid hormone levels are at the root of abnormalities in glucose homeostasis due to their effects on endogenous glucose production and IR. It is well known that IR is one of the main prerequisites for hyperglycemia during pregnancy [33].

In our previous study, a relationship between thyroid dysfunction during pregnancy and gestational diabetes was observed [19].

As already mentioned, universal screening for thyroid dysfunction during pregnancy is not widely accepted. Measurement of the thyroid hormones levels is usually recommended in pregnant women who are undergoing therapy due to thyroid disease or are aware of the existence of pathology of the latter without treatment.

The time for screening for carbohydrate disorders is often delayed, and there are already complications for both the mother and the developing fetus.

Searching for and finding biomarkers that could serve as predictors of the manifestation of these endocrine disorders during pregnancy may be effective in preventing adverse maternal and fetal outcomes.

### Limitations

Our study has several limitations. First, the study is monocentric, covering a relatively small population sample. Second, the analysis adopts a retrospective design. Further prospective studies with multiple monitoring of PlGF levels during pregnancy, in parallel with thyroid hormones levels testing and performing OGTT in each trimester, are underway to confirm these results. In this case, the serum levels of PlGF in different stages of pregnancy will be assessed, and accordingly, its predictive value concerning the manifestation of carbohydrate and thyroid disorders will be re-analyzed.

## 5. Conclusions

A major aspect of modern medicine is prevention. Both thyroid dysfunction and GDM can lead to adverse maternal and fetal outcomes. Our analysis shows that patients with registered GDM and thyroid dysfunction during pregnancy had higher MoM PlGF levels compared to the control group. It is clear that PlGF MoM above 0.8 may serve as an additional recommendation for both OGTT and thyroid function screening in early pregnancy.

## Figures and Tables

**Table 1:** Maternal characteristics and biochemical parameters of observed women.

Maternal Characteristics	GDM Group (*n* = 412)	Control Group (*n* = 250)	*p*-Value
Maternal age (years)	33.3 (4.9)	32.8 (4.3)	0.251
BMI (kg/cm^2^)	26.1 (5.3)	22.9 (5.8)	0.422
FPG (mmol/L)	5.5 (0.8)	4.8 (0.7)	0.001
TSH (mIU/L)	2.5 (1.4)	2.46 (0.8)	0.001
FT4 (pmol/L)	13.3(2.6)	14.2(3.1)	0.001
FT3 (pmol/L)	4.1 (0.8)	3.9 (0.7)	0.019
PlGF MoM	0.9 (0.7–1.2)	0.7 (0.6–0.8)	0.001

Abbreviations: BMI, body mass index; FPG, fasting plasma glucose GDM, gestational diabetes mellitus; FT3, triiodothyronine; FT4, free thyroxine; TSH, thyroid-stimulating hormone levels; PlGF, placental growth factor; MoM, multiple of the median. Note: Data are expressed as mean (standard deviation) or median (IQR).

**Table 2:** Sensitivity, specificity, cutoff value, and area under the curve (AUC) of PlGF in the prediction and diagnosis of GDM.

Variable	Sensitivity (%)	Specificity (%)	Cutoff	AUC (95% CI)	*p*-Value
PlGF	51.2	87.2	0.89	0.68 (0.64–0.72)	<0.001

**Table 3:** Sensitivity, specificity, cutoff value, and area under the curve (AUC) of PlGF in the prediction and diagnosis of thyroid dysfunction.

Variable	Sensitivity (%)	Specificity (%)	Cutoff	AUC (95% CI)	*p*-Value
PlGF	58.2	62.9	0.81	0.61 (0.57–0.65)	<0.001

## Data Availability

All data presented in this study are available from the corresponding author upon reasonable request.
